# Crocin promotes ferroptosis in gastric cancer via the Nrf2/GGTLC2 pathway

**DOI:** 10.3389/fphar.2025.1527481

**Published:** 2025-03-21

**Authors:** Nan Yan, Gaofu Li, Linglin Zhao, Qijing Guo, Jie Yang, Jianhong Liu, Wei Zhou, Yue Gao, Yushuang Luo

**Affiliations:** 1 Research Center for High Altitude Medicine, Key Laboratory of High Altitude Medicine (Ministry of Education), Key Laboratory of High Altitude Medicine in Qinghai Province, Qinghai Province Plateau Medicine Applied and Basic Research Key Laboratory (Qinghai-Utah Plateau Medicine Joint Key Laboratory), Qinghai University, Xining, China; 2 Department of Pharmaceutical Sciences, Beijing Institute of Radiation Medicine, Beijing, China; 3 Department of oncology, Air force medical center. PLA, Beijing, China; 4 Affiliated Hospital of Qinghai University, Xining, Qinghai, China; 5 College of Humanities and Technology, QingHai Open University, Xining, China; 6 State Key Laboratory of Kidney Diseases, Chinese PLA General Hospital, Beijing, China

**Keywords:** gastric cancer, crocin, GGTLC2, ferroptosis, Nrf2

## Abstract

**Introduction:** Gastric cancer (GC) is characterized by high incidence and poor survival rates. Crocin, a natural carotenoid from saffron, exhibits antioxidant, anti-inflammatory, and anti-tumor properties. Ferroptosis, an iron-dependent cell death driven by lipid peroxidation, plays a critical role in cancer progression and is a potential therapeutic target. This study investigates whether crocin inhibits GC cell proliferation by inducing ferroptosis and explores its underlying mechanisms.

**Methods:** This study employed in vivo and in vitro models to assess crocin’s effects on GC cell proliferation, apoptosis, migration, invasion, and ferroptosis. Pathway enrichment analysis was performed on differentially expressed genes post-crocin treatment. Lentiviral vectors were used to knockdown and overexpress GGTLC2, exploring its role in GC progression and crocin’s therapeutic effects. The UCSC and JASPAR databases predicted Nrf2 binding sites in the GGTLC2 promoter. Molecular docking evaluated crocin’s affinity for Nrf2 and GGTLC2. Immunofluorescence and nuclear-cytoplasmic fractionation assays analyzed Nrf2 expression and localization. ChIP-qPCR determined Nrf2’s regulatory role on GGTLC2 and crocin’s modulatory effects.

**Results:** The results demonstrated that crocin significantly inhibited the proliferation, migration, and invasion of GC cells while promoting apoptosis. Differentially expressed genes following crocin treatment were predominantly enriched in pathways associated with oxidative stress and ferroptosis. Crocin downregulated the oncogene GGTLC2, thereby suppressing GC cell proliferation, invasion, and migration, while simultaneously promoting apoptosis and ferroptosis. Molecular docking analysis revealed a stable binding affinity between crocin and GGTLC2, suggesting that crocin may directly target GGTLC2 to modulate its expression. Additionally, crocin facilitated the translocation of Nrf2 from the nucleus to the cytoplasm. ChIP-qPCR results confirmed that Nrf2 directly binds to the GGTLC2 promoter region to regulate its expression, and crocin attenuated this binding interaction.

**Discussion:** In conclusion, our findings suggest that crocin, as a promising natural compound for GC therapy, may inhibit ferroptosis in GC cells through the Nrf2/GGTLC2 signaling pathway, thereby suppressing tumor initiation and progression. This study provides novel insights into the molecular mechanisms underlying the anti-tumor effects of crocin and highlights its potential as a therapeutic agent for GC.

## 1 Introduction

According to the 2020 Global Cancer Statistics, gastric cancer (GC) is identified as the fifth most prevalent cancer globally and the fourth leading cause of cancer-related mortality, surpassed only by lung, colorectal, and liver cancers (Yang WJ. et al., 2023). The early symptoms of GC are frequently non-specific, and the current diagnostic and treatment options remain substantially limited, resulting in a high proportion of cases being diagnosed at advanced stages (Wagner et al., 2017). Chemotherapy, although widely utilized as a primary treatment for advanced GC, is associated with suboptimal efficacy, significant side effects, and a high risk of resistance, collectively contributing to a markedly low 5-year survival rate among affected patients (Patel and Cecchini, 2020; Liu et al., 2022). Previous investigations have demonstrated that the combined treatment of crocin and cisplatin significantly enhances the inhibition and apoptosis of GC cells compared to cisplatin monotherapy, thereby suggesting the potential utility of crocin as an adjunctive agent to improve chemotherapeutic outcomes (Luo et al., 2017). Consequently, crocin is posited as a promising herbal compound with the capacity to improve the efficacy of conventional chemotherapy regimens for GC. Nevertheless, the precise molecular mechanisms underlying the anti-proliferative and pro-apoptotic effects of crocin remain to be elucidated, representing a critical area for future research.

Crocin, the primary bioactive constituent of saffron, demonstrates significant anti-inflammatory and antioxidant properties (Bastani et al., 2022). Numerous studies have shown that crocin exhibits antitumor activity in breast, colorectal, prostate, and gastric cancers (Lambrianidou et al., 2020). Research by Xia et al. revealed that the antitumor effects of crocin in human colorectal cancer cell lines may be mediated by p53 expression (Xia, 2015). Chen et al. demonstrated that crocin suppresses lung adenocarcinoma cell proliferation by upregulating p53, inducing cell cycle arrest and apoptosis, and increasing sensitivity to chemotherapeutic agents such as cisplatin and pemetrexed (Chen et al., 2015). Zhou et al. further elucidated that crocin inhibits the migration, invasion, and epithelial-mesenchymal transition of GC cells through the miR-320/KLF5/HIF-1α signaling pathway (Zhou et al., 2019). Additionally, Hoshyar et al. reported that crocin promotes apoptosis in gastric adenocarcinoma cells by increasing the Bax/Bcl-2 ratio and activating caspases (Hoshyar et al., 2013).

Ferroptosis, a regulated form of iron-dependent cell death, is characterized by the accumulation of intracellular iron, elevated reactive oxygen species (ROS) production, and the promotion of apoptotic pathways (Yan et al., 2023). The heightened proliferative activity of cancer cells significantly increases their iron requirements, resulting in elevated oxidative stress levels. This renders cancer cells particularly vulnerable to ferroptosis, thereby positioning it as a promising therapeutic target (Murray and Dixon, 2024). Nuclear factor erythroid 2-related factor 2 (Nrf2 or NFE2L2), an antioxidant transcription factor, plays a pivotal role in regulating ferroptosis in cancer cells. It achieves this by modulating various pathways, including iron metabolism, the expression of the metabolic gene glutathione peroxidase 4 (GPX4), and key downstream molecules involved in Nrf2-mediated oxidative stress responses (Jiang et al., 2024). Recent studies by Li et al. have demonstrated that Nrf2 transcriptionally activates AKR1B1 expression in GC, thereby inhibiting ferroptosis and promoting cancer cell survival (Li X. et al., 2024). Furthermore, several natural products have been identified as potential inducers of ferroptosis through the Nrf2 pathway (Yuhao et al., 2024). For instance, triptolide has been shown to promote ferroptosis by suppressing Nrf2 nuclear translocation, thereby overcoming doxorubicin resistance in leukemia cells (Wu et al., 2023). Despite these advancements, the potential interplay between crocin, Nrf2, and ferroptosis remains unexplored, highlighting a critical gap in current research.

Gamma-glutamyl transferase (GGT), an enzyme localized to the cell membrane, is essential for maintaining glutathione homeostasis, redox balance, and detoxification processes (Heisterkamp et al., 2008). Aberrant expression of the GGT family has been documented in a wide range of malignancies (Bui et al., 2015; Bansal et al., 2019; Mottaghitalab et al., 2021; Zhao et al., 2024). GGTLC2, a constituent of the GGT family, has been characterized as an antioxidant gene, with emerging evidence indicating a potential link between its expression levels and sarcoma patient outcomes (Quan et al., 2022). Nevertheless, the functional significance of GGTLC2 in GC remains unexplored.

The present study endeavors to clarify the impacts of crocin on the incidence and progression of GC, along with the molecular mechanisms underlying these effects. The overarching goal is to provide a solid theoretical and experimental foundation to support the potential clinical utilization of crocin in the treatment of GC.

## 2 Materials and methods

### 2.1 Cell culture

The human GC cell lines AGS, HGC27, and MKN45 were procured from the Cell Resource Center at Peking Union Medical College (PUMC), while the GES-1 cell line was obtained from FuHeng Biology (Shanghai, China). Cells were maintained at 37°C in a humidified atmosphere containing 5% CO_2_, using culture medium supplemented with 10% fetal bovine serum (FBS) and 1% penicillin/streptomycin.

### 2.2 Public datasets

RNA-Seq expression data for stomach adenocarcinoma (STAD) were downloaded from The Cancer Genome Atlas (TCGA) database (https://www.cancer.gov/ccg/research/genome-sequencing/tcga) in transcripts per million (TPM) format. The dataset comprised 448 samples. Differential expression analysis of GGTLC2 was performed using the “ggplot2” and “ggpubr” packages in R 4.3.1, while survival analysis and Kaplan-Meier (KM) curves were generated using the “survival” and “survminer” packages.

### 2.3 Cytotoxicity assay

The cytotoxic effects of crocin (Sigma-Aldrich, #17304) were evaluated using the Cell Counting Kit-8 (CCK-8; E1008, APPLYGEN). Cells were seeded at a density of 5,000 cells per well in 96-well plates and treated with crocin at concentrations ranging from 5 μM to 10 mM for 24 h. Following treatment, 10 µL of CCK-8 reagent was added to each well, and plates were incubated for 1 h at 37°C. Absorbance was measured at 450 nm using a Cytation™ 5 microplate reader (BioTek). The half-maximal inhibitory concentration (IC50) was calculated using GraphPad Prism 9 and utilized for subsequent experiments.

### 2.4 Construction of GGTLC2 overexpression and silencing cell line

The lentiviral vector for GGTLC2 overexpression (pHBLV-CMV-MCS-EF1-ZsGreen-T2A-puro) was procured from Hanbang Biotechnology (Shanghai, China). The cloning primers were designed as follows: sense, 5′-act​aga​gga​tct​att​tcc​ggt​gaa​ttc​gcc​acc​ATG​ACC​TCT​GAG​TTC-3'; antisense, 5′-cag​atc​ctt​act​agt​atc​gat​gga​tcc​tca​GTA​GCC​GGC​AGG​CTC​CCC​G-3'. For GGTLC2 knockdown, the shRNA lentivirus vector GV493 was purchased from Genechem (Shanghai, China), with target sequences shRNA1: 5′-CCT​GTT​CAA​TGA​TGA​AAT​GGA-3′ and shRNA2: 5′-AGC​GAG​ATC​CTG​TTC​AAT​GAT-3'. Lentivirus transfection was conducted following the manufacturer’s instructions. Stable cell lines were selected using puromycin (2 μg/mL) 48 h post-transfection. Transfection efficiency was validated through fluorescence microscopy, quantitative reverse transcription polymerase chain reaction (qRT-PCR), and Western blot (WB) analysis.

### 2.5 Cell proliferation

Cell proliferation was evaluated using the Cell Counting Kit-8 (E1008, APPLYGEN). Cells were seeded at a density of 3 × 10^4^ cells/mL (100 μL/well) in a 96-well plate. Following 24 h of crocin treatment, 10 µL of CCK-8 reagent was added to each well, and plates were incubated at 37°C in a 5% CO_2_ atmosphere for 1 h. Absorbance was measured at 450 nm using a Cytation™ 5 microplate reader (BioTek, Winooski, Vermont, United States).

### 2.6 Immunofluorescence

Immunofluorescence was employed to assess Ki-67 expression and Nrf2 localization. Cells were cultured in confocal dishes (NEST, 801002) until 70%–80% confluence was achieved. Following 24 h of crocin treatment (where applicable), cells were fixed with 1 mL of immunostaining fixative (Beyotime, P0098) for 10 min at room temperature and washed twice with immunostaining wash solution (Beyotime, P0106). Blocking was performed using immunostaining blocking solution (Beyotime, P0102) for 60 min at room temperature. Primary antibodies, anti-Ki-67 (Abcam, ab16667) and anti-Nrf2 (Abcam, ab137550), were diluted 1:250 in immunostaining primary antibody dilution solution (Beyotime, P0103) and incubated at 4°C for 12 h. The secondary antibody, goat anti-rabbit IgG H&L (Alexa Fluor^®^ 647) (Abcam, ab150079), was diluted 1:500 in immunofluorescence secondary antibody dilution solution (Beyotime, P0108) and incubated at room temperature in the dark for 60 min. Nuclei were stained with DAPI (Beyotime, P0131) for 10 min. Images were acquired using the BioTek Cytation 5 and Gen5 software (Agilent, Santa Clara, California). Ki-67 fluorescence intensity was quantified using ImageJ.

### 2.7 Flow cytometry detection of cell apoptosis

Apoptosis was assessed using the Annexin V-PE/7-AAD Apoptosis Detection Kit (Yeasen Biotech, 40310ES60) for lentivirus-transfected cells and the Annexin V-FITC Kit (Beyotime, C1062 L) for non-fluorescently labeled cells. Cells were digested with trypsin (without EDTA), centrifuged, and resuspended in Binding Buffer at a density of 1 × 10^6^ cells/mL. Subsequently, 100 μL of cell suspension was mixed with 5 μL Annexin V-PE (or Annexin V-FITC) and 5 μL 7-AAD (or PI), followed by incubation in the dark at room temperature for 15 min. After adding 400 μL Binding Buffer, samples were analyzed using a flow cytometer.

### 2.8 Wound healing assays

Cells were cultured in 12-well plates until reaching 80%–90% confluence. A uniform scratch was created using a sterile pipette tip, and cells were treated with crocin for 24 h. Wound closure was monitored by capturing images at 0 and 24 h using a 4× optical microscope, and quantitative analysis was performed using Photoshop and ImageJ.

### 2.9 Transwell invasion assays

Transwell chambers (8.0 μm pore size; Corning, 3422) were coated with Matrigel matrix (Corning, 354234) (1:6 dilution in serum-free medium) and incubated at 37°C for 30 min. Cells in the logarithmic growth phase were resuspended in serum-free medium at a density of 1 × 10^5^ cells/mL, and 200 μL of cell suspension was added to the upper chamber. The lower chamber was filled with 600 μL complete medium supplemented with 10% FBS. After 24 h of incubation, non-invading cells were removed, and invading cells were fixed with 4% paraformaldehyde, stained with crystal violet, and counted under an inverted microscope. Three random fields were analyzed for each sample using ImageJ.

### 2.10 Functional enrichment analysis of the DEGs

In the initial exploratory study, we employed the Affymetrix GeneChip PrimeView human gene expression microarray to comprehensively evaluate the gene expression patterns in human GCcells (AGS). Cells were treated with 2.45 mM crocin to investigate its effects on the transcriptomic landscape of GC cells (Luo et al., 2020). The “ggplot2” and “ggpubr” packages in R were used to visualize GGTLC2 expression profiles. Gene Ontology (GO) and Kyoto Encyclopedia of Genes and Genomes (KEGG) pathway enrichment analyses were performed using the clusterProfiler package, and results were visualized with ggplot2.

### 2.11 Lipid peroxidation levels

Lipid peroxidation levels were quantified using the BODIPY 581/591 C11 detection kit. According to the manufacturer’s instructions, BODIPY 581/591 C11 was diluted to 2 μM for the preparation of the staining solution. Cells were seeded in confocal dishes and treated with crocin for 24 h upon reaching 70%–80% confluence. After removing the culture medium, the BODIPY 581/591 C11 working solution was added, and cells were incubated in the dark at 37°C with 5% CO_2_ for 30 min. Cells were then washed twice with PBS and observed under a fluorescence microscope at excitation wavelengths of 488 nm (oxidized state) and 581 nm (reduced state). Fluorescence intensity was quantified using ImageJ to determine the green-to-red fluorescence ratio.

### 2.12 Glutathione (GSH) assay

Processed cells were harvested, washed with PBS, and treated with protein removal reagent M. Cells were subjected to two freeze-thaw cycles and lysed on ice for 5 min. The supernatant was collected by centrifugation. For total GSH detection, detection buffer, enzyme solution, and color reagent were mixed. A 50 µL aliquot of the standard and sample was added to each well of a 96-well plate, followed by 150 µL of working solution. After incubation in the dark at 37°C for 5 min, absorbance was measured at 412 nm. For GSSG detection, samples were mixed with GSH scavenger and reacted at room temperature for 60 min. The working solution was prepared, and 50 µL of the standard and treated sample was added to each well, followed by 150 µL of working solution. After incubation in the dark at 37°C for 5 min, 50 µL of 0.5 mg/mL NADPH solution was added, and absorbance was measured at 412 nm. GSH and GSSG concentrations were calculated based on the standard curve, with GSH concentration determined by subtracting GSSG from total GSH.

### 2.13 Ferrous iron (Fe^2+^) level assay

The intracellular Fe^2+^ concentration was quantified using FerroOrange (DOJINDO, F374). A 10 µM working solution of FerroOrange was prepared according to the manufacturer’s instructions. Cells were seeded in confocal dishes and cultured to 70%–80% confluence. After 24 h of crocin treatment, cells were washed twice with HBSS buffer, and the FerroOrange working solution was added. Cells were incubated in the dark at 37°C with 5% CO_2_ for 30 min. Fluorescent images were captured using BioTek Cytation 5 and Gen5 software, and fluorescence intensity was measured with ImageJ.

### 2.14 Quantitative reverse transcription polymerase chain reaction (qRT-PCR)

Total RNA was extracted using TransZol Up reagent (TransGen Biotech, ET111-01) according to the manufacturer’s protocol. cDNA was synthesized using the TransScript One-Step gDNA Removal and cDNA Synthesis SuperMix (TransGen Biotech, AT311-04). Real-time PCR was performed using PerfectStart Green qPCR SuperMix (TransGen Biotech, AQ601-01-V2) with the following cycling conditions: 30 s at 94°C, followed by 40 cycles of 5 s at 94°C, 15 s at 57°C, and 10 s at 72°C. Primers were synthesized by Sangon Bioengineering (Shanghai) Co., Ltd. The GGTLC2 primers used were Forward 5′-ACT​GCA​CTG​ATA​TGT​GTC​ACC​C-3′ and Reverse 5′-AGC​CGA​ACC​AGA​GGT​TGT​AGA-3′. GAPDH was amplified using the primers Forward 5′-CTG​GGC​TAC​ACT​GAG​CAC​C-3′ and Reverse 5′-AAG​TGG​TCG​TTG​AGG​GCA​ATG-3′. Relative gene expression was calculated using the 2^−ΔΔCT^ method and normalized to GAPDH.

### 2.15 Western blot (WB)

Cellular proteins were isolated using RIPA lysis buffer supplemented with protease and phosphatase inhibitors. Protein concentration was determined using the bicinchoninic acid (BCA) protein assay kit (Applygen Technologies, Beijing, China). Xenograft tumor tissues were minced, flash-frozen, and pulverized. After adding lysis buffer, the homogenate was incubated on ice for 30 min, centrifuged at 12,000 rpm (4°C, 15 min), and the supernatant was collected. Protein concentration was determined using a BCA assay kit. Approximately 30 µg of proteins were separated by SDS-PAGE and transferred to a PVDF membrane (IPVH00010, Millipore, Germany) at 100 mA for 150 min. Membranes were blocked with Quick Block Buffer (P1623, Applygen Technologies, Beijing, China) for 30 min at room temperature before incubation with primary antibodies: Rabbit polyclonal antibody to GGTLC2 (Biorbyt, orb513965), Anti-Bcl2 (Abcam, ab182858), Anti-Bax (Abcam, ab32503), Anti-FACL4 (Abcam, ab205197), Anti-GPX4 (Abcam, ab125066), Anti-Nrf2 (CST, 12,721), Anti-Lamin B1 (Abcam, ab16048), Anti-GAPDH (Abcam, ab9485), and Anti-Beta Actin (Abcam, ab8226). After washing, membranes were incubated with goat anti-rabbit IgG H&L (HRP) (Abcam, ab6721) and goat anti-mouse IgG H&L (HRP) (Abcam, ab205719). Protein expression was visualized using a Millipore chemiluminescence device, and band intensities were quantified using ImageJ.

### 2.16 Animal experiments

Animal experiments were approved by the Animal Care and Use Committee of the Affiliated Hospital of Qinghai University and conducted in compliance with the Declaration of Helsinki guidelines. Five-week-old female Balb/c nude mice were obtained from Vital River (Beijing, China). After acclimatization, mice were randomly divided into two groups (Vector, OE; n = 12 per group). To establish a subcutaneous tumor model, MKN45 cells were mixed with Matrigel (1:1 ratio) at 4°C to a concentration of 5 × 10^7^ cells/mL. A 200 μL aliquot of the cell suspension was injected subcutaneously into the right shoulder of each mouse. Tumors were observed after 7 days, and mice were further divided into subgroups (Vehicle, Crocin; n = 6 per subgroup). Crocin (100 mg/kg, 10 μL/g) was administered via intraperitoneal injection every other day, while the control group received saline. Tumor size and mouse weight were measured every other day. When tumors reached 1 cm in length, mice were euthanized, and tumors were excised and weighed. Tumor volume was calculated as 1/2 × length × width^2^.

### 2.17 Histological analysis

Tumor tissues were excised from the animal model and fixed in 4% paraformaldehyde for 24 h. Tissues were embedded in paraffin, dehydrated, and sectioned for H&E staining to evaluate tissue morphology.

### 2.18 Immunohistochemistry

Antigen retrieval was performed using pH 6 sodium citrate buffer, followed by blocking with 1% bovine serum albumin (Beyotime, ST025). Samples were incubated overnight at 4°C with anti-Ki-67 antibody (Abcam, ab16667; 1:200 dilution). Slides were then incubated with goat anti-rabbit IgG H&L (HRP polymer) (Abcam, ab214880; 1:200 dilution) and stained using the DAB Horseradish Peroxidase Color Development Kit (Beyotime, P0203). Images were captured using an inverted microscope and analyzed with ImageJ Pro Plus 6.0.

### 2.19 Molecular docking

Compound structures were obtained from PubChem and converted to PDB format using Open Babel 2.3.2. Receptor proteins were retrieved from UniProt, and water molecules and ligands were removed using PyMOL 2.3.4. In AutoDockTools, hydrogen atoms were added, and charges were balanced. Both receptor and ligand were converted to PDBQT format. Molecular docking was performed using AutoDock Vina 1.1.2, and results were visualized in PyMOL.

### 2.20 Cytosol-nuclei fractionation

Cytoplasmic and nuclear proteins were separated using a nuclear-cytosol extraction kit (Applygen, P1200) according to the manufacturer’s protocol. Cells were seeded and treated with crocin for 24 h at 70%–80% confluence. Cells were resuspended in pre-chilled cell lysis buffer A and incubated on ice for 10–15 min. After centrifugation, the supernatant (cytoplasmic fraction) was collected, and the pellet (nuclear fraction) was resuspended in pre-chilled cell lysis buffer B. The nuclear fraction was incubated on ice for 30 min with intermittent vortexing. After centrifugation, the nuclear supernatant was collected. Fractions were analyzed by WB.

### 2.21 Chromatin immunoprecipitation (ChIP)

Transcription factors upstream of GGTLC2 were predicted using the UCSC database, and potential binding sites were identified using JASPAR. ChIP was performed using the SimpleChIP^®^ Plus Enzymatic Chromatin IP Kit (Cell Signaling Technology, 9,004) according to the manufacturer’s protocol. qPCR primers were Forward, 5′-GGC​CCC​TCT​TCC​ACT​TTC​G-3'; Reverse, 5′-AGC​TCG​TGC​CCC​AAA​TGA​A-3'.

### 2.22 Statistical analysis

Data were analyzed using one-way ANOVA for multiple comparisons and Student’s t-test for group comparisons. Results are expressed as mean ± standard deviation from at least three independent experiments. The Wilcoxon test was used to compare GGTLC2 expression between GC and normal tissues. Statistical analysis was performed using GraphPad Prism 9.5.1, with significance set at p < 0.05.

## 3 Manuscript formatting

### 3.1 Crocin inhibits GC cell proliferation, migration, and invasion, promoting apoptosis

**FIGURE 1:**
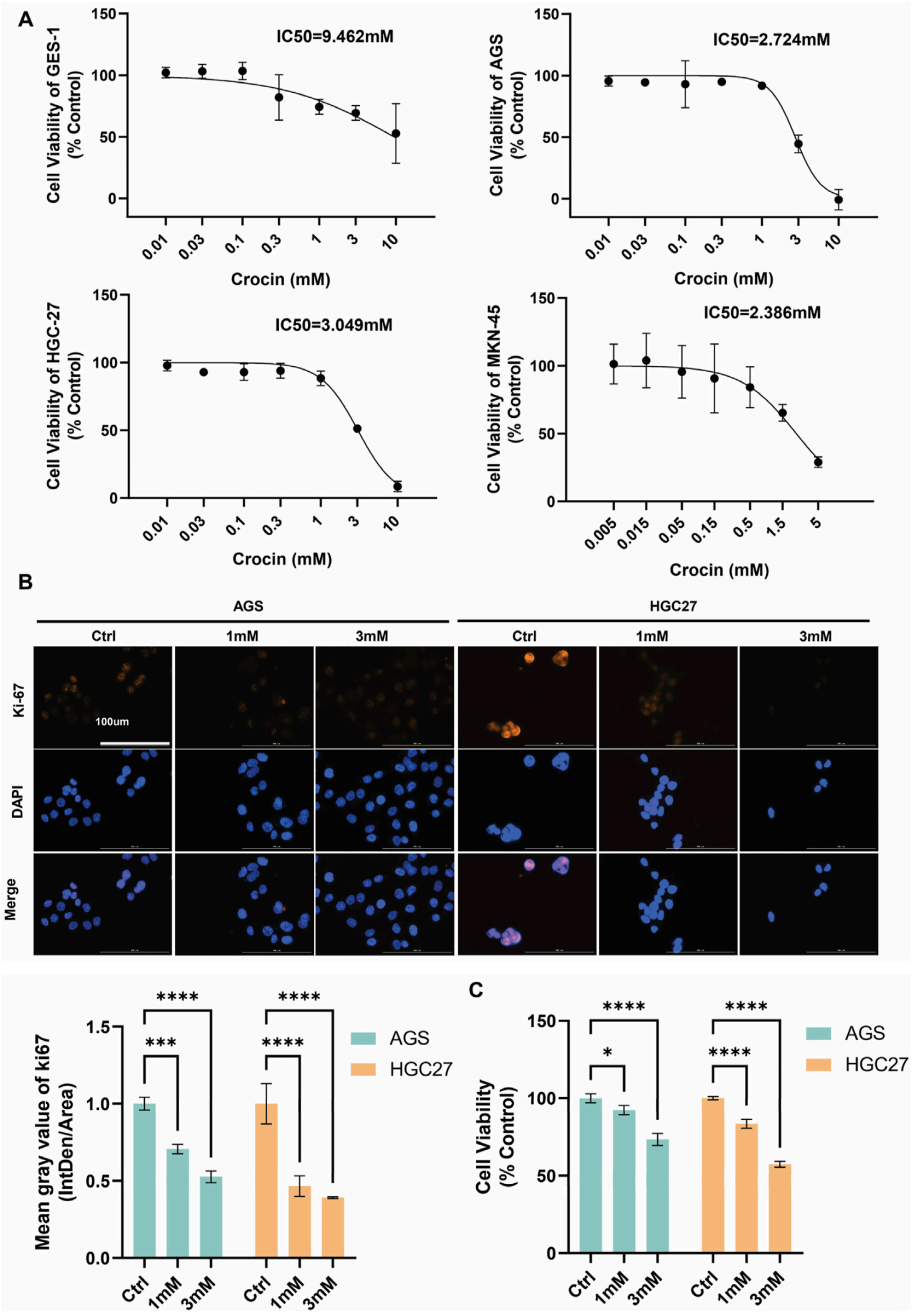
Crocin inhibits the proliferation of GC cells **(A)** The IC50 values of GES1, AGS, HGC27, and MKN45 following treatment with crocin. **(B)** Immunofluorescence detection revealed that, following crocin treatment, the expression of Ki-67 decreased in a concentration-dependent manner. **(C)** The CCK-8 analysis indicates that treating the cells with crocin at low (1 mM) and high (3 mM) concentrations results in a concentration-dependent decrease in cell viability. *p < 0.05, **p < 0.01, ***p < 0.001, and ****p < 0.0001. “ns” indicates no significa.

The sensitivity of GC cell lines (AGS, HGC27, MKN45) and GES1 cells to crocin (0.005–10 mM) was evaluated. Cells treated with culture medium served as controls. MKN45, a semi-adherent cell line, exhibited the highest sensitivity to crocin (IC50 = 2.386 mM), followed by AGS (IC50 = 2.824 mM) and HGC27 (IC50 = 3.049 mM). Notably, crocin showed limited cytotoxicity in GES1 cells (IC50 = 9.462 mM) (Figure 1A). These results indicate that crocin inhibits GC cell growth with minimal toxicity to normal gastric mucosal epithelial cells. AGS and HGC27 were selected for *in vitro* studies based on their IC50 values, while MKN45 was chosen for *in vivo* experiments due to its tumorigenic properties.

**FIGURE 2:**
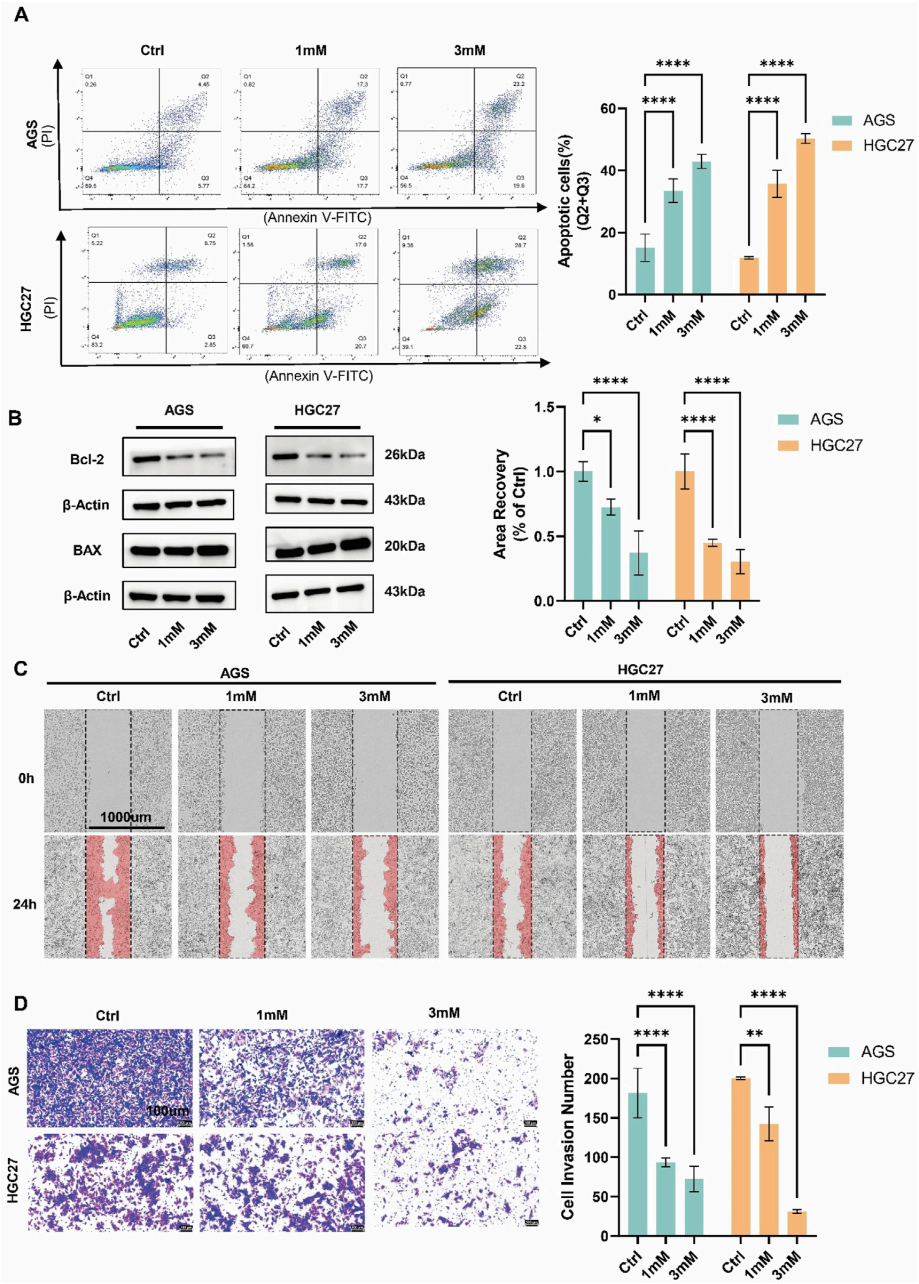
Crocin extract inhibits the migration, and invasion of GC cells while promoting apoptosis **(A)** The results of flow cytometry indicated that the number of apoptotic cells increased in a concentration-dependent manner following treatment with both low (1 mM) and high (3 mM) concentrations of crocin. **(B)** WB demonstrated a concentration-dependent reduction in Bcl-2 levels alongside a corresponding dose-dependent upregulation of BAX following crocin treatment across both low and high concentration ranges. **(C)** Wound healing studies indicate that treatment with both l ow (1 mM) and high (3 mM) concentrations of crocin results in a concentration-dependent decrease in the cell migration rates of AGS and HGC27 cells. **(D)** Transwell results indicate that treatment with both low (1 mM) and high (3 mM) of crocin leads to a concentration-dependent decrease in the number of invasive cells in the AGS and HGC27 cell lines. *p < 0.05, **p < 0.01, ***p < 0.001, and ****p < 0.0001. “ns” indicates no significa.

To investigate the effects of crocin on proliferation, migration, invasion, and apoptosis, AGS and HGC27 cells were treated with low (1 mM) and high (3 mM) concentrations of crocin. CCK-8 and immunofluorescence assays revealed that, following crocin treatment, the expression level of Ki-67 and cell viability decreased in a concentration-dependent manner, indicating that crocin inhibits the growth of AGS and HGC27 cells (Figures 1B, C). Flow cytometry and WB analysis were employed to assess the impact of crocin on apoptosis in GC cells. WB analysis demonstrated a concentration-dependent reduction in Bcl-2 levels alongside a corresponding dose-dependent upregulation of BAX following crocin treatment across both low and high concentration ranges (Figure 2B). Since Bcl-2 is an anti-apoptotic protein and BAX is a pro-apoptotic protein, the decrease in Bcl-2 and increase in BAX directly prove that crocin promotes apoptosis in GC cells. Flow cytometry results demonstrated a significant increase in apoptotic cells in a concentration-dependent manner (Figure 2A), further supporting this conclusion. Wound healing assays showed that crocin concentration-dependently inhibited the migration of AGS and HGC27 cells (Figure 2C). Furthermore, Transwell assays indicated that crocin concentration-dependently downregulated the invasive capacity of the cells (Figure 2D). Collectively, these results suggest that crocin inhibits the proliferation, migration, and invasion of GC cells while promoting apoptosis.

### 3.2 Crocin decreased the levels of GPX4 and GSH, reduced lipid peroxidation, and increased Fe^2+^ accumulation, thereby inducing ferroptosis in GC cells

**FIGURE 3:**
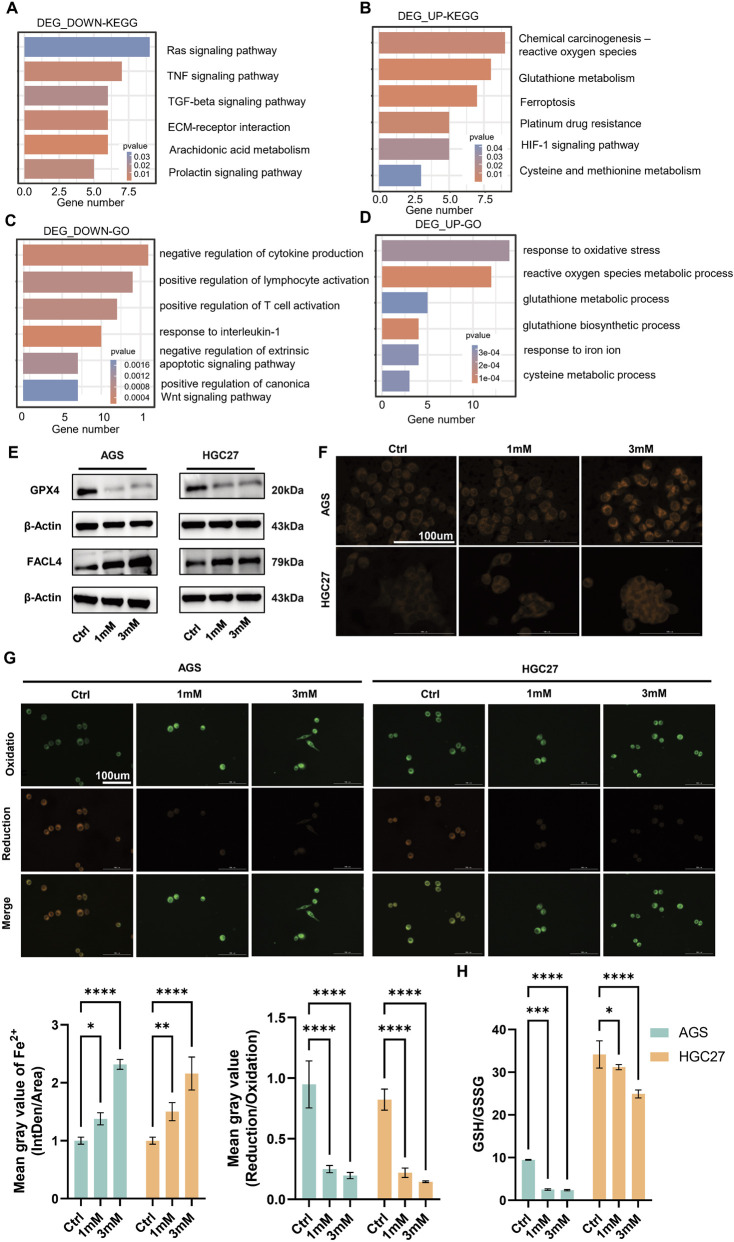
Crocin induces ferroptosis in GC cells **(A)** KEGG pathways enriched with downregulated genes following crocin treatment. **(B)** KEGG pathways enriched with upregulated genes following crocin treatment. **(C)** The results of GO analysis for downregulated genes following crocin treatment. **(D)** The results of GO analysis for upregulated genes following crocin treatment. **(E)** WB analysis revealed that crocin treatment significantly downregulated GPX4 expression while concurrently upregulating FACL4 levels. **(F)** After treatment with crocin, the accumulation of Fe2+ increases in a concentration-dependent manner. **(G)** After treatment with crocin, the levels of lipid peroxidation decrease in a concentration-dependent manner. **(H)** After treatment with crocin, the GSH/GSSG ratio demonstrates a concentration-dependent decrease. *p < 0.05, **p < 0.01, ***p < 0.001, and ****p < 0.0001. “ns” indicates no significa.

Gene expression and functional enrichment were analyzed using the Affymetrix GeneChip PrimeView array after crocin treatment (2.45 mM). A total of 216 genes were upregulated and 301 genes were downregulated (Supplementary Table S1). IPA revealed significant enrichment of differentially expressed genes in the NRF2-mediated oxidative stress response pathway (Luo et al., 2020). KEGG and GO analyses showed that downregulated genes were enriched in the “Ras signaling pathway,” “TNF signaling pathway,” and “TGF-beta signaling pathway,” while upregulated genes were enriched in “Chemical carcinogenesis-reactive oxygen species,” “Glutathione metabolism,” “Ferroptosis,” and “Platinum drug resistance” (Figures 3A, B). GO analysis indicated that downregulated genes were enriched in “negative regulation of cytokine production,” “positive regulation of T cell activation,” and “negative regulation of extrinsic apoptotic signaling pathway,” while upregulated genes were enriched in “response to oxidative stress,” “glutathione metabolic process,” and “response to iron ion” (Figures 3C, D). These results suggest that crocin inhibits GC growth by inducing ferroptosis. Further analysis showed that crocin decreased GPX4, GSH, and lipid peroxidation levels, while increasing FACL4 and Fe^2+^ levels in a concentration-dependent manner (Figures 3E–H). These findings indicate that crocin promotes ferroptosis in GC cells.

### 3.3 Crocin inhibits the proliferation, migration, and invasion of GC cells and promotes apoptosis and ferroptosis by downregulating GGTLC2, both *in vivo* and *in vitro*

**FIGURE 4:**
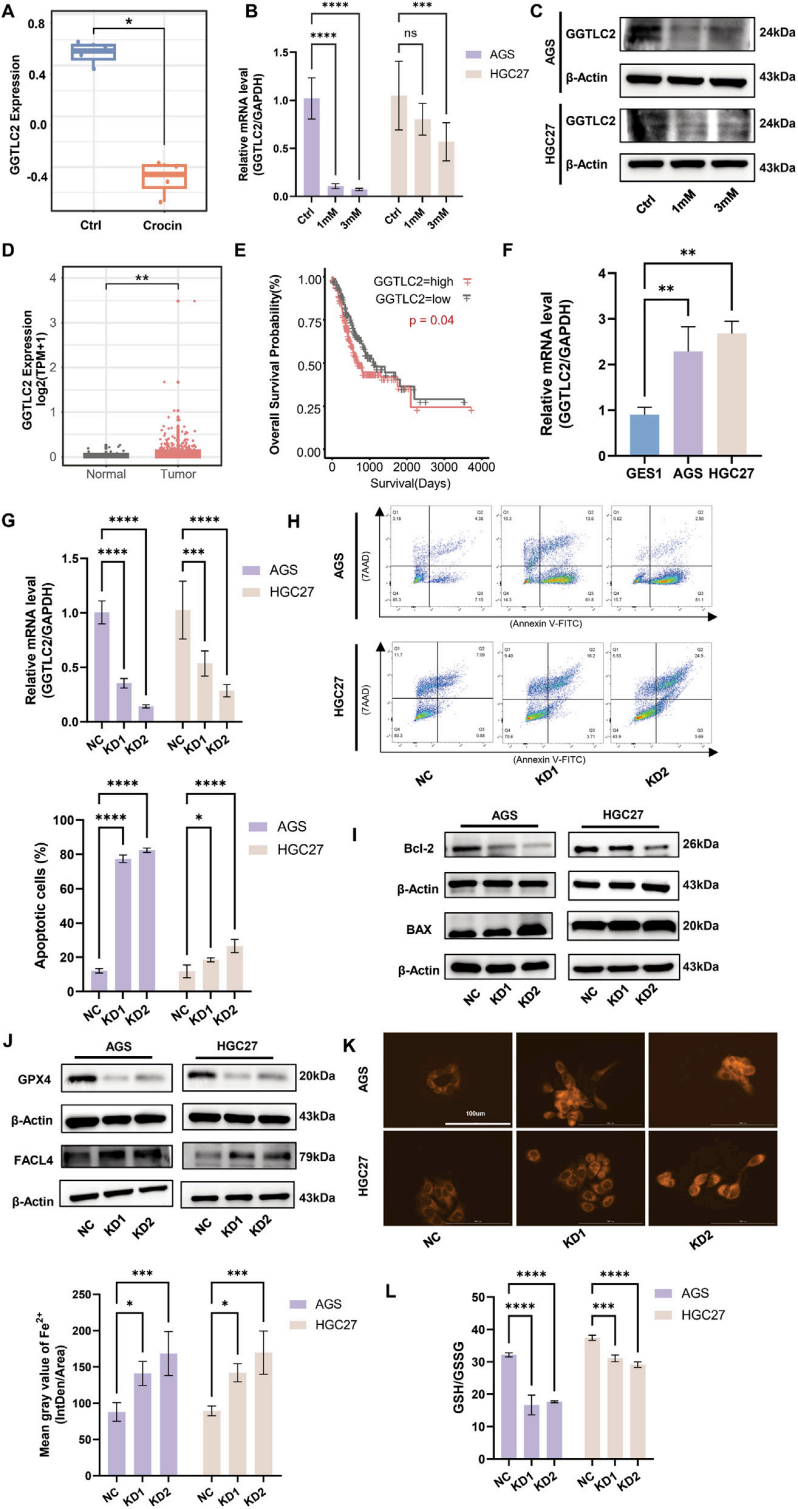
GGTLC2 is highly expressed in GC and is associated with a poor prognosis. **(A)** Gene chip analysis indicates that GGTLC2 expression is downregulated in the crocin treatment group compared to the control group. **(B)** The qPCR results indicate that the expression of GGTLC2 is downregulated following treatment with crocin. **(C)** The WB results indicate that the GGTLC2 level is downregulated following treatment with crocin. **(D)** The analysis of data from the TCGA database indicates that the expression level of GGTLC2 is downregulated in GC tissues. **(E)** Survival analysis shows that high GGTLC2 expression correlates with a poor prognosis. **(F)** qPCR results indicate that GGTLC2 is highly expressed in AGS and HGC27 compared to GES1. **(G)** Knockdown of GGTLC2 was achieved through lentiviral transfection, followed by validation using qPCR. **(H)** Flow cytometry revealed an increase in apoptotic cells following GGTLC2 knockdown. **(I)** WB analysis demonstrated that GGTLC2 knockdown significantly downregulated Bcl-2 expression while concurrently upregulating BAX levels. **(J)** WB analysis demonstrated that GGTLC2 knockdown significantly downregulated GPX4 expression while concurrently upregulating FACL4 levels. **(K)** After knocking down GGTLC2, the expression of Fe2+ is upregulated. **(L)** After knocking down GGTLC2, the expression of GSH/GSSG is reduced. *p < 0.05, **p < 0.01, ***p < 0.001, and ****p < 0.0001. “ns” indicates no significa.

Preliminary gene chip analysis revealed that GGTLC2 expression was downregulated following treatment with crocin (Figure 4A). Cellular experiments, including qPCR and WB, confirmed this concentration-dependent downregulation (Figures 4B, C). GGTLC2, a member of the GGT family, has been demonstrated in previous studies to play a significant role in the prevention and diagnosis of sarcomas (Quan et al., 2022). Currently, no research has investigated the correlation between GGTLC2 and GC. Using the TCGA database, the present study analyzed the expression and prognostic significance of GGTLC2 in GC. The results indicated that GGTLC2 was highly expressed in GC tissues and was associated with patient prognosis (Figures 4D, E). Cellular experiments showed that, compared with the GES-1 cell line, the expression level of GGTLC2 was significantly elevated in the AGS and HGC27 cell lines (Figure 4F).

To clarify GGTLC2’s impact on apoptosis and ferroptosis in GC cells, we constructed a GGTLC2-knockdown lentivirus. After transfection, qPCR results demonstrated a significant reduction in GGTLC2 expression in AGS and HGC27 cells (Figure 4G). Flow cytometry (Figure 4H) and WB analysis (Figure 4I) revealed that GGTLC2 knockdown led to a significant elevation in apoptotic cells, downregulated Bcl-2 expression, and concurrently upregulated BAX levels, suggesting that GGTLC2 knockdown promoted apoptosis in GC cells. Having established the role of GGTLC2 knockdown in promoting apoptosis, we next investigated its relationship with ferroptosis in GC cells. We assessed the levels of Fe^2+^, GSH, FACL4, and GPX4 in GC cells after GGTLC2 knockdown. The results demonstrated that, following GGTLC2 knockdown, the levels of GPX4 and GSH underwent a reduction (Figures 4J, L), while FACL4 and Fe^2+^ levels were significantly elevated (Figures 4J, K). These findings suggest that GGTLC2 knockdown inhibits the development and progression of GC by promoting ferroptosis in GC cells. The reduction in GPX4 and GSH levels, along with the elevation of FACL4 and Fe^2+^ levels, are hallmarks of ferroptosis. Thus, the observed changes indicate that GGTLC2 knockdown likely activates the ferroptotic pathway, thereby impeding the growth and spread of GC cells.

**FIGURE 5:**
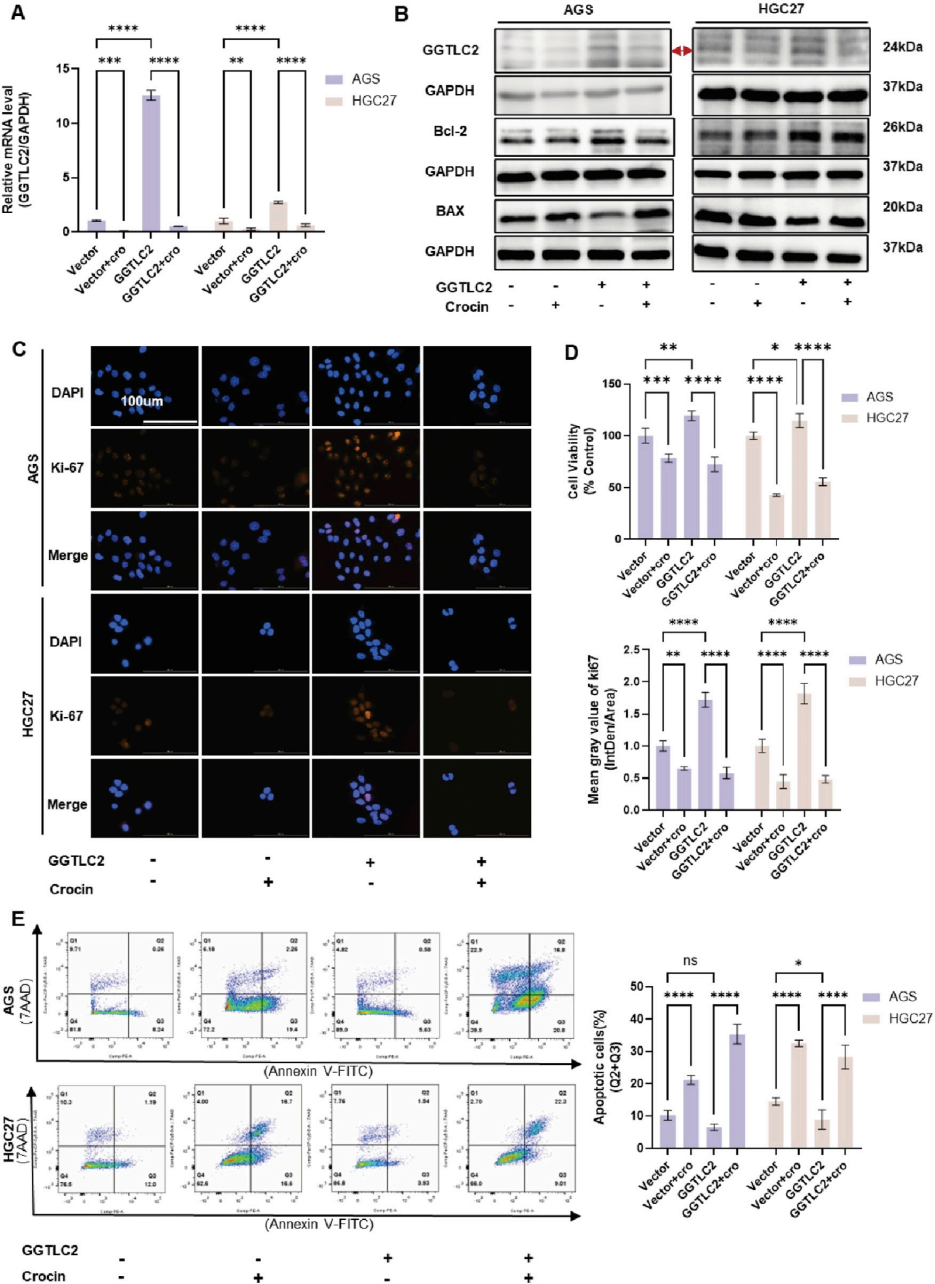
Crocin inhibits the proliferation of GC cells and promotes apoptosis by targeting GGTLC2 **(A)** qPCR results indicate that GGTLC2 is highly expressed in the OE group compared to the Vector group, but is downregulated in both groups after crocin treatment (at IC50 concentration). **(B)** WB results showed that compared with Vector group, the levels of GGTLC2 and Bcl-2 in OE group were increased, but the levels of GGTLC2 and Bcl-2 in both groups were decreased after crocin treatment. In contrast, BAX levels were downregulated in the OE group compared to the Vector group, but increased in both groups after crocin treatment. **(C)** Immunofluorescence results indicate that Ki-67 expression is higher in the OE group compared to the Vector group; however, it is downregulated in both groups following crocin treatment. **(D)** CCK-8 results indicate higher cell viability in the OE group compared to the Vector group, but crocin treatment reduces viability in both groups. **(E)** Flow cytometry results indicate that the number of apoptotic cells is lower in the OE group compared to the Vector group; however, the number of apoptotic cells increases in both groups following crocin treatment. *p < 0.05, **p < 0.01, ***p < 0.001, and ****p < 0.0001. “ns” indicates no significa.

**FIGURE 6:**
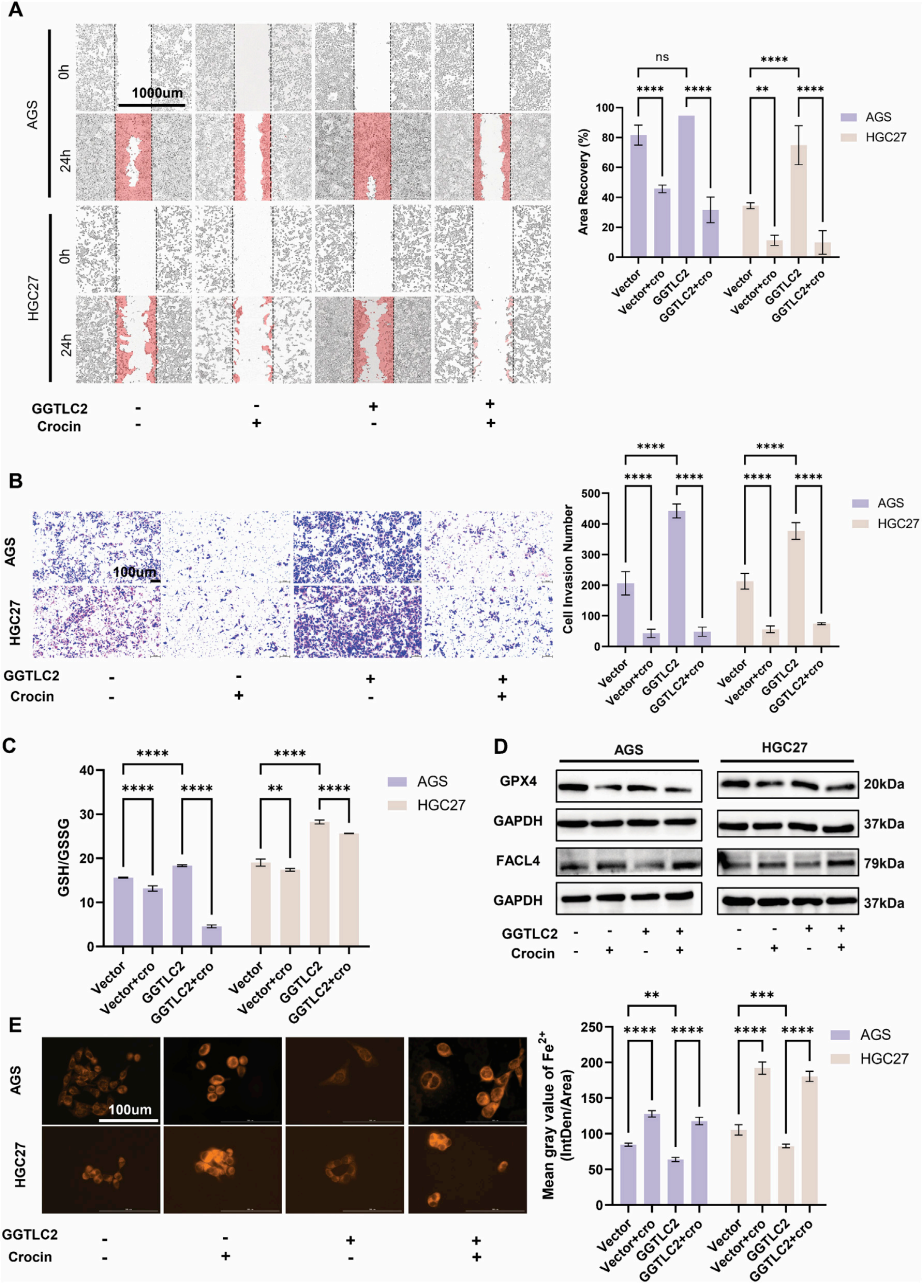
Crocin inhibits the invasion and migration of GC cells by targeting GGTLC2 and promotes ferroptosis. **(A)** Wound healing assays indicate that the OE group’s migration rate increased relative to the Vector group, but both groups decreased after crocin treatment (at IC50 concentration). **(B)** Transwell results indicate that the number of invasive cells in the OE group is significantly increased compared to the Vector group, which is reduced after crocin treatment. **(C)** Compared to the Vector group, the GSH/GSSG ratio in the OE group is significantly elevated; after crocin treatment, the GSH/GSSG ratios in both groups are downregulated. **(D)** WB analysis revealed that the OE group exhibited higher basal GPX4 expression than the Vector group, but crocin treatment downregulated GPX4 in both groups. Conversely, FACL4 expression was lower in the OE group at baseline, while crocin treatment upregulated FACL4 in both groups. **(E)** The OE group exhibits lower levels of Fe2+ compared to the Vector group; however, crocin treatment increased Fe2+ accumulation in both groups. *p < 0.05, **p < 0.01, ***p < 0.001, and ****p < 0.0001. “ns” indicates no significa.

To further elucidate the regulatory effect of crocin on GGTLC2, a lentivirus for GGTLC2 overexpression (OE) was developed. After transfection, WB and qPCR analyses demonstrated a significant increase in GGTLC2 expression in AGS and HGC27 cells. In addition, AGS and HGC27 cells overexpressing GGTLC2 were treated with crocin at its IC50 concentration. The results indicated that GGTLC2 expression was downregulated following crocin treatment (Figures 5A, B). CCK-8 assays and immunofluorescence analyses revealed that the expression of Ki-67 and cell viability were significantly elevated in GGTLC2-overexpressing cells; however, this upward trend was markedly inhibited following crocin treatment (Figures 5C, D). Flow cytometry results showed a significant reduction in both early and late apoptotic cells after GGTLC2 overexpression, while crocin treatment led to a notable increase in apoptotic cells. WB results demonstrated that compared with the Vector group, the levels of Bcl-2 in the OE group were increased, but the levels of Bcl-2 in both groups were decreased after crocin treatment. In contrast, BAX levels were downregulated in the OE group compared to the Vector group, but increased in both groups after crocin treatment (Figure 5B). This suggests that GGTLC2 overexpression inhibits apoptosis in GC cells, whereas crocin downregulates this inhibitory effect, thereby promoting apoptosis (Figure 5E). Wound healing assays showed that GGTLC2 overexpression enhanced AGS and HGC27 cell migration, while crocin inhibited it (Figure 6A). Additionally, Transwell assays demonstrated that GGTLC2 overexpression increased AGS and HGC27 cell invasion, whereas crocin inhibited it (Figure 6B).

Previous research by our group indicated that crocin inhibits the development and progression of GC by promoting ferroptosis in GC cells. To further elucidate the mechanism of action of crocin, the expression levels of Fe^2+^, GSH, FACL4 and GPX4 in GC cells overexpressing GGTLC2 following crocin treatment were investigated. The results showed that, after the overexpression of GGTLC2, the levels of GSH and GPX4 were upregulated; however, this increasing trend was significantly suppressed after crocin treatment. Conversely, FACL4 expression was lower in the OE group, while crocin treatment upregulated FACL4 in both groups (Figures 6C, D). Additionally, overexpression of GGTLC2 reduced Fe^2+^ levels in GC cells, while crocin treatment significantly increased them (Figure 6E).

**FIGURE 7:**
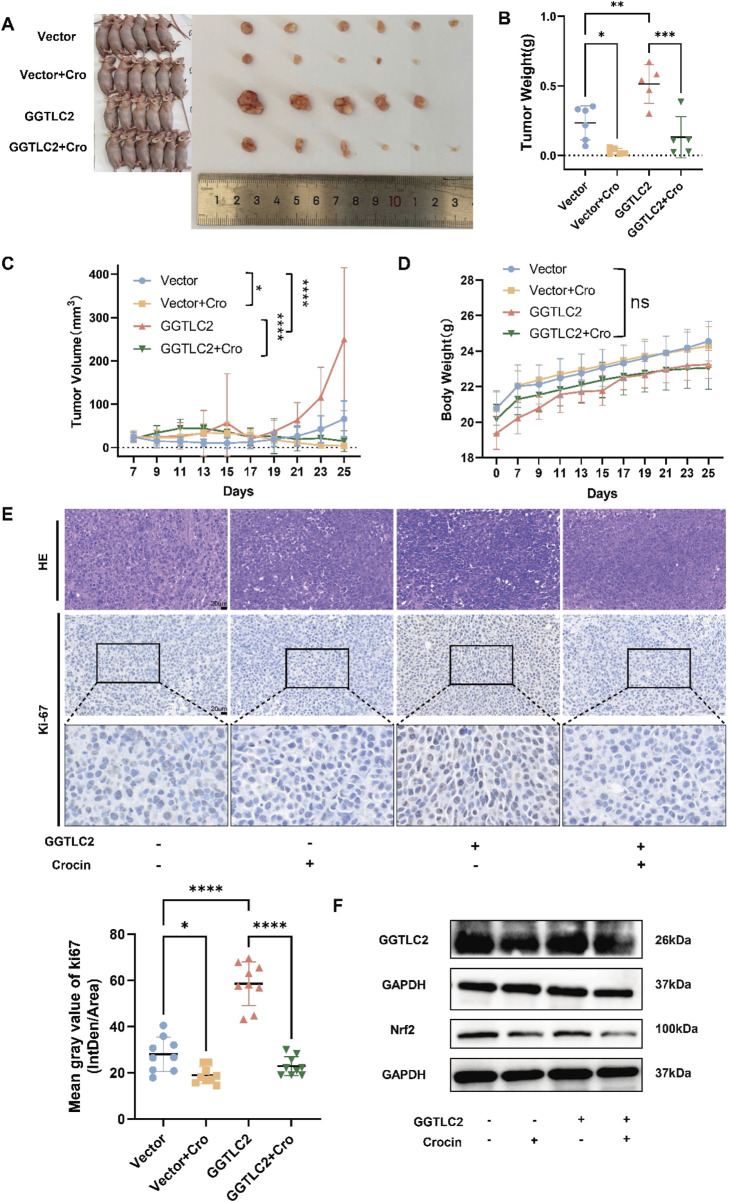
Crocin targets GGTLC2 to inhibit the proliferation of GC cells *in vivo* **(A)** Compared to the control group, the size of xenograft tumors in the GGTLC2 overexpression group of nude mice was significantly increased, while treatment with crocin significantly reduced tumor size *in vivo*. **(B)** The GGTLC2 overexpression group of nude mice had significantly heavier xenograft tumors than the control group, while crocin treatment significantly reduced tumor weight *in vivo*. **(C)** Compared to the control group, the tumor volume in the GGTLC2 overexpression group was significantly increased, while treatment with crocin significantly reduced tumor volume *in vivo*. **(D)** There was no significant difference in the body weight of nude mice among the vector group, the GGTLC2 overexpression group, and the crocin treatment group. **(E)** The immunohistochemical results indicate that, compared to the Vector group, the GGTLC2 overexpression group exhibited a significant increase in Ki-67 levels, while after treatment with crocin, the Ki-67 levels significantly decreased. **(F)** WB analysis showed that GGTLC2 levels were significantly higher in the GGTLC2 OE group than in the Vector group. Crocin treatment, however, significantly reduced GGTLC2 expression in both groups. Although baseline Nrf2 levels did not differ significantly between the OE and Vector groups, crocin treatment markedly downregulated Nrf2 in both groups. *p < 0.05, **p < 0.01, ***p < 0.001, and ****p < 0.0001. “ns” indicates no significa.

Based on the *in-vitro* findings, *in-vivo* experiments were then conducted to further validate the role of crocin and GGTLC2 in GC. *In vivo* experiments demonstrated that tumor size and weight significantly increased in the GGTLC2 overexpression group compared to the vector group, while the crocin treatment group exhibited a significant reduction (Figures 7A–C). Body weight remained consistent across all four groups (Figure 7D). Immunohistochemical analysis revealed elevated Ki-67 expression in the overexpression group, which was significantly inhibited by crocin treatment (Figure 7E). Additionally, the expression profiles of GGTLC2 and Nrf2 in xenograft tumor tissues were assessed. WB analysis demonstrated that under basal conditions, GGTLC2 levels were significantly elevated in the OE group compared to the Vector group. Following crocin treatment, however, a marked reduction in GGTLC2 expression was observed in both groups. Notably, although baseline Nrf2 expression did not differ significantly between the OE and Vector groups, crocin administration led to a pronounced downregulation of Nrf2 in both experimental groups (Figure 7F).

In summary, crocin inhibits the proliferation, migration, and invasion of GC cells by promoting apoptosis and ferroptosis through the downregulation of GGTLC2.

### 3.4 Crocin inhibits the nuclear translocation of Nrf2 and inhibits the onset and development of GC through the Nrf2/GGTLC2 pathway

**FIGURE 8:**
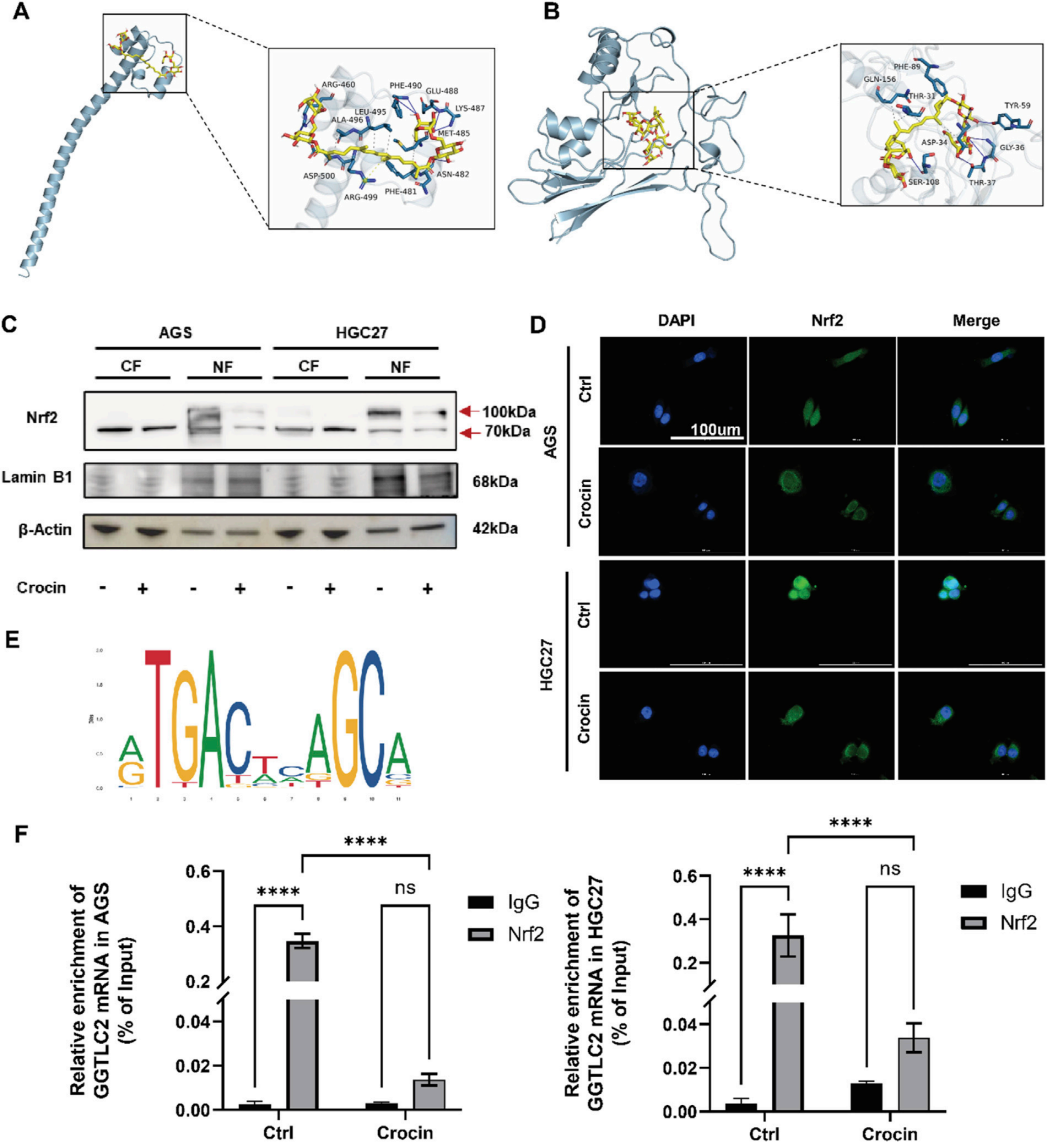
Crocin inhibits the onset and progression of GC through the Nrf2/GGTLC2 pathway **(A)** The molecular docking results indicate a stable binding interaction between crocin and GGTLC2. **(B)** The molecular docking results indicate a stable binding interaction between crocin and Nrf2. **(C)** The WB results indicate that after treatment with crocin (at IC50 concentration), the expression of Nrf2 in the cell nucleus is significantly downregulated (CF: Cytoplasmic Fraction; NF: Nuclear Fraction). **(D)** The immunofluorescence results indicate that following crocin treatment, Nrf2 translocates from the nucleus to the cytoplasm. **(E)** The potential binding sites between Nrf2 and the GGTLC2 promoter were identified using the JASPAR database. **(F)** ChIP-qPCR results show that Nrf2 binds to the GGTLC2 promoter to regulate its expression, while crocin inhibits this binding. *p < 0.05, **p < 0.01, ***p < 0.001, and ****p < 0.0001. “ns” indicates no significa.

The individual components of traditional Chinese medicine are complex and diverse, and their mechanisms of action often exhibit characteristics of multiple targets and pathways. At the protein level, as depicted in Figure 4C, the expression of the GGTLC2 protein is downregulated following crocin treatment. To elucidate the underlying mechanism, we employed molecular docking to explore the binding mode between crocin and GGTLC2. The results indicated a stable binding between crocin and GGTLC2, with a free energy of −7.4 kcal/mol (Figure 8A).

At the transcriptional level, components of traditional Chinese medicine can regulate transcription factors’ expression, activity, or subcellular localization, thus influencing the transcription of target genes (Zhang et al., 2021). Our previous research indicated that differentially expressed genes are significantly enriched in the Nrf2-mediated oxidative stress response following crocin treatment. Given the effect of crocin on GGTLC2, we further explored its impact on Nrf2, a crucial transcription factor related to cellular antioxidant responses. Molecular docking analysis using AutoDock Vina showed a stable binding affinity between crocin and Nrf2 (free energy of −6.9 kcal/mol, Figure 8B), indicating that crocin may directly target Nrf2 to modulate its expression and activity. Additionally, immunofluorescence results demonstrated that after crocin treatment, Nrf2 shifted from the nucleus to the cytoplasm, indicating the inactivation of its transcriptional function (Figure 8D). Nuclear and cytoplasmic proteins were isolated to assess crocin’s impact on Nrf2 expression in both the cytoplasmic and nuclear compartments. The WB results revealed that, following crocin treatment, the expression of Nrf2 in the nucleus was significantly downregulated, which is consistent with previous immunofluorescence findings (Figure 8C). The WB analysis of Nrf2 revealed two prominent bands at approximately 70 kDa and 100 kDa. The 70 kDa band corresponds to the unmodified Nrf2 protein, while the 100 kDa band is likely a post-translationally modified form.

Nrf2 is a crucial transcription factor associated with the regulation of cellular antioxidant responses. To further determine whether the inhibition of GGTLC2 expression by crocin is associated with the transcriptional repression of Nrf2, we first utilized the UCSC database to predict potential transcription factors that may bind to GGTLC2. The results indicated that Nrf2 (Score: 414, p < 10^−4^) has the potential to bind to the GGTLC2 promoter. Additionally, we employed JASPAR to predict the binding sites of Nrf2 on the GGTLC2 promoter and identified possible binding sites between Nrf2 and the GGTLC2 promoter (NC_000022.11:22642614–22644614 AGGACTCAGCC) (Figure 8E), which were further validated with ChIP-qPCR experimental results. The ChIP-qPCR results demonstrated that Nrf2 directly binds to the promoter region of GGTLC2 to regulate its expression, while crocin inhibits this binding effect (Figure 8F).

Therefore, our findings suggest that crocin promotes the translocation of the Nrf2 protein from the nucleus to the cytoplasm, thereby inhibiting the transcription and expression of GGTLC2. Crocin may inhibit the occurrence and progression of GC through the Nrf2/GGTLC2 pathway.

## 4 Discussion

GC is characterized by a high incidence rate, insidious onset, and poor prognosis (Li et al., 2022). Our previous research demonstrated that the combination of crocin and cisplatin significantly inhibits the proliferation of GC cells to a greater extent than cisplatin alone (Luo et al., 2020; Li Y. et al., 2024). This study investigated the effects of crocin on GC incidence and progression and explored its potential mechanisms. We discovered, for the first time, that crocin may inhibit the occurrence of ferroptosis in GC cells via the Nrf2/GGTLC2 pathway.

Ferroptosis is an iron-dependent, lipid peroxidation-induced non-apoptotic cell death modality that occurs upon disruption of intracellular redox homeostasis (Yan et al., 2023). Ferroptosis regulation is highly complex, with GPX4 being the most crucial negative regulator in this process. GPX4 uses reduced GSH as a substrate to convert toxic lipid hydroperoxides into non-toxic lipid alcohols, directly or indirectly inhibiting lipid peroxidation and thus suppressing ferroptosis (Yang et al., 2014).

Nrf2 is a well-characterized antioxidant transcription factor (Murray and Dixon, 2024). Studies have demonstrated that Nrf2 exerts a dual effect in tumors: it aids in cancer prevention in normal cells but confers a protective advantage in tumor cells when over-activated (Hu et al., 2024). Nrf2 may promote resistance to ferroptosis in cancer cells through various pathways. Nrf2 may enhance cancer cell resistance to ferroptosis through multiple pathways. Xiaofang Sun et al. showed that nuclear Nrf2 activates genes related to heme, iron, and ROS metabolism, such as NQO1, HO1, and FTH1, contributing to ferroptosis resistance (Sun et al., 2016). The labile iron pool (LIP) is a crucial factor associated with ferroptosis (Dixon et al., 2012). Anandhan A et al. reported that Nrf2 inhibition in ovarian cancer leads to an increase in LIP, enhancing cell sensitivity to ferroptosis (Anandhan et al., 2023). Notably, one major mechanism by which Nrf2 inhibits ferroptosis is through upregulating GSH synthesis. GPX4 is an anti-ferroptotic molecule, and GSH serves as its substrate. Therefore, increasing GSH levels can prevent ferroptosis (Murray and Dixon, 2024). Yang Z et al. identified that in GC, the oncogene ACTL6A, in concert with Nrf2, transcriptionally regulates the expression of the γ-glutamyl-cysteine ligase catalytic subunit (GCLC), promoting GSH synthesis and inhibiting ferroptosis (Yang Z. et al., 2023). This study identified that GC occurrence and progression may be associated with the transcriptional activation of the GGT family gene GGTLC2 by Nrf2.

GGT is a protease anchored to the cell membrane, consisting of a light chain and a heavy chain. The light chain is extracellular and exhibits enzymatic activity, while the heavy chain has a transmembrane structure that anchors the protein to the cell membrane (Corti et al., 2010). GGT plays a pivotal role in extracellular GSH metabolism (Griffith et al., 1978). GGT cleaves extracellular GSH into cysteine-glycine (Cys-Gly) and glutamate via the γ-glutamyl cycle pathway. Subsequently, Cys-Gly is degraded by dipeptidase into cysteine and glycine, which are transported into the cell to participate in GSH synthesis (Jan et al., 2013). Cysteine concentration is the primary limiting factor for glutathione synthesis efficiency. The elevated metabolic rate in tumors leads to increased ROS production, which raises the demand for GSH in tumor cells. GGT overexpression in tumors enhances cysteine uptake by cancer cells, resulting in increased intracellular GSH levels and thus inhibiting oxidative stress-triggered cell death pathways (Ganesaratnam et al., 2004; Giovambattista et al., 2010; Pompella et al., 2022). In cancer cells under oxidative stress, GGT overexpression activates a pathway enabling cells to acquire additional cysteine sources. This mechanism contributes to maintaining intracellular GSH levels and enhancing drug-resistance (Hanigan, 2014). GGTLC2, a protein-coding gene belonging to the GGT family, is closely associated with GGT’s biological functions (Nora et al., 2008). It encodes the light chain of GGT, which exhibits catalytic activity. This study aimed to investigate the critical role of GGTLC2 in GC. Our study demonstrated that GGTLC2 is highly expressed in GC, facilitating cell proliferation, invasion, and migration, and inhibiting apoptosis and ferroptosis.

Crocin, the main active component of saffron, is a water-soluble carotenoid with established antioxidant and anti-inflammatory properties. In cancer cells, it exerts anti-tumor effects through multiple mechanisms, such as promoting apoptosis, inhibiting proliferation, invasion, and metastasis, enhancing chemotherapy sensitivity, and improving immune function (Bao et al., 2023). In this study, it was observed that crocin at concentrations of 2–10 mM *in vitro* and 100 mg/kg *in vivo* effectively inhibited the proliferation of GC. An attempt was made to compare these doses with the serum concentrations achievable in humans following oral administration. However, the absorption, distribution, metabolism, and excretion (ADME) of crocin in humans remain incompletely understood. Several studies have indicated that the oral bioavailability of crocin is significantly low, rendering it difficult to directly extrapolate our experimental doses to clinically relevant serum concentrations in humans. For instance, research by Almodóvar et al. demonstrated that the concentration of crocin isomers significantly decreased during the digestive process, with a bioaccessibility of only 40.77% *in vitro*. *In vivo* pharmacokinetic studies involving 13 healthy adult volunteers administered two different doses (56 mg and 84 mg) of commercial saffron tablets revealed that crocin was undetectable in the plasma of participants. This can likely be explained by the susceptibility of crocin to degradation under physiological conditions (37°C and varying pH levels) in the stomach and intestines, leading to limited bioavailability and insufficient absorption across the intestinal epithelium (Almodóvar et al., 2020). Given the limited pharmacokinetic data on crocin in humans and its low bioavailability following oral administration, it is currently not feasible to directly compare the high doses used in our *in vitro* and *in vivo* experiments with the serum concentrations achievable in humans through oral intake. Significantly, several studies have explored the use of nanocarriers, such as lipid-based or polymeric nanoparticles, to deliver crocin, which has been demonstrated to enhance its stability and preserve its therapeutic efficacy (Rahaiee et al., 2017; Soltani et al., 2024). In the future, the development of novel formulations or delivery systems may improve the absorption and stability of crocin, thereby optimizing its bioavailability. These considerations are critical for translating our preclinical findings into clinically relevant therapeutic strategies.

The mechanism of crocin’s action may be associated with lipid metabolism pathways. Hashemi et al. reported that saffron extract could inhibit breast cancer progression by reducing cholesterol and triglyceride levels in the serum of tumor-bearing mice and in breast cancer cell lines (Hashemi et al., 2020). The active components of traditional Chinese medicine are complex and diverse, and their mechanisms of action often feature multiple targets and pathways. This study aimed to elucidate the potential mechanisms through which crocin influences the expression of GGTLC2 at both the protein and transcriptional levels. At the protein level, monomers from traditional Chinese medicine can directly bind to target proteins, affecting their spatial structure, active sites, or interactions with other molecules, thus altering protein function. For example, they can inhibit enzyme catalytic activity by binding to the active site or block signaling pathways by attaching to receptors (Han et al., 2023; Sheng et al., 2023; Zhang et al., 2025). Crocin exhibits a strong and stable binding affinity with GGTLC2. GGTLC2 encodes the light chain of GGT, an enzyme composed of a light chain and a heavy chain. The heavy chain is responsible for enzyme localization and membrane anchoring, while the light chain has catalytic activity, facilitating gamma-glutamyl group transfer and participating in glutathione metabolism and cellular antioxidant processes. Thus, we hypothesize that crocin’s direct targeting of GGTLC2 may be related to its enzymatic activity. Furthermore, we explored the mechanism through which crocin impacts GGTLC2 at the transcriptional level. At this level, monomers from traditional Chinese medicine can interact with transcription factors. Transcription factors, a class of proteins, bind to specific gene promoter regions, regulating gene transcription initiation. These monomers can modulate transcription factor expression, activity, or subcellular localization, thus influencing the transcriptional processes of target genes (Zheng et al., 2023). Crocin promotes the translocation of Nrf2 from the nucleus to the cytoplasm, resulting in its inactivation and inhibiting the transcription of the oncogene GGTLC2. Consequently, the proliferation, invasion, migration, and ferroptosis of GC cells are suppressed. Our findings link crocin, the antioxidant transcription factor Nrf2, and the lipid peroxidation-induced ferroptosis pathway, offering new insights into the anticancer mechanisms of crocin.

## 5 Conclusion

This study investigates the molecular mechanisms by which crocin inhibits the onset and progression of GC. The findings indicate that crocin suppresses GC proliferation, invasion, and migration by downregulating the oncogene GGTLC2 while simultaneously promoting apoptosis and ferroptosis. Furthermore, crocin facilitates the translocation of the transcription factor Nrf2 from the nucleus to the cytoplasm. Nrf2 can directly bind to the promoter region of GGTLC2 to regulate its expression, and crocin inhibits this binding effect. Therefore, we propose that crocin, as a promising herbal monomer for GC treatment, may inhibit GC cell ferroptosis through the Nrf2/GGTLC2 pathway, thereby suppressing the occurrence and progression of GC.

## Data Availability

The raw data supporting the conclusions of this article will be made available by the authors, without undue reservation.
