# Correction: Crocin promotes ferroptosis in gastric cancer via the Nrf2/GGTLC2 pathway

**DOI:** 10.3389/fphar.2026.1901759

**Published:** 2026-07-10

**Authors:** Nan Yan, Gaofu Li, Linglin Zhao, Qijing Guo, Jie Yang, Jianhong Liu, Wei Zhou, Yue Gao, Yushuang Luo

**Affiliations:** 1 Research Center for High Altitude Medicine, Key Laboratory of High Altitude Medicine (Ministry of Education), Key Laboratory of High Altitude Medicine in Qinghai Province, Qinghai Province Plateau Medicine Applied and Basic Research Key Laboratory (Qinghai-Utah Plateau Medicine Joint Key Laboratory), Qinghai University, Xining, China; 2 Department of Pharmaceutical Sciences, Beijing Institute of Radiation Medicine, Beijing, China; 3 Department of oncology, Air Force Medical Center. PLA, Beijing, China; 4 Affiliated Hospital of Qinghai University, Xining, Qinghai, China; 5 College of Humanities and Technology, QingHai Open University, Xining, China; 6 State Key Laboratory of Kidney Diseases, Chinese PLA General Hospital, Beijing, China

**Keywords:** gastric cancer, crocin, GGTLC2, ferroptosis, Nrf2

In the original published version of this article, inadvertent figure assembly errors were identified in Western blot images (Figure 3E, Figure 4J) and wound-healing micrographs (Figure 6A), as detailed below.

**FIGURE 3:**
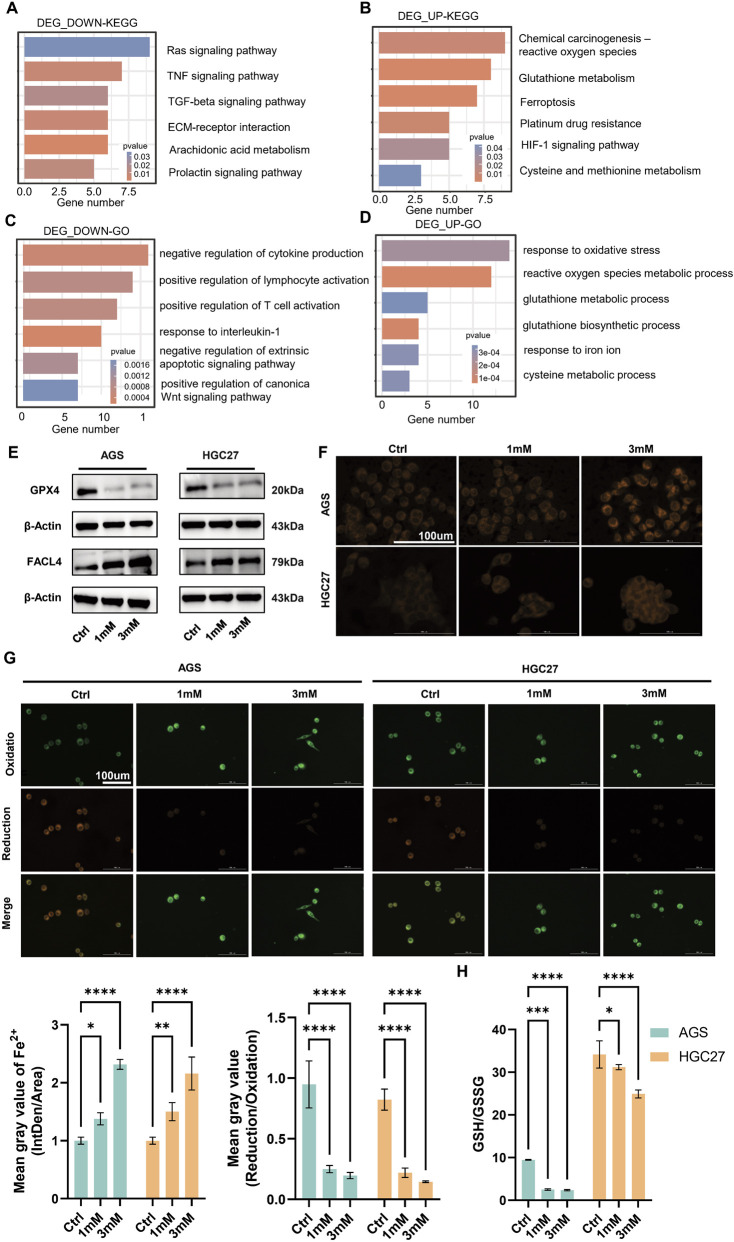
Crocin induces ferroptosis in GC cells **(A)** KEGG pathways enriched with downregulated genes following crocin treatment. **(B)** KEGG pathways enriched with upregulated genes following crocin treatment. **(C)** The results of GO analysis for downregulated genes following crocin treatment. **(D)** The results of GO analysis for upregulated genes following crocin treatment. **(E)** WB analysis revealed that crocin treatment significantly downregulated GPX4 expression while concurrently upregulating FACL4 levels. **(F)** After treatment with crocin, the accumulation of Fe2+ increases in a concentration-dependent manner. **(G)** After treatment with crocin, the levels of lipid peroxidation decrease in a concentration-dependent manner. **(H)** After treatment with crocin, the GSH/GSSG ratio demonstrates a concentration-dependent decrease. *p < 0.05, **p < 0.01, ***p < 0.001, and ****p < 0.0001. “ns” indicates no significa.

**FIGURE 4:**
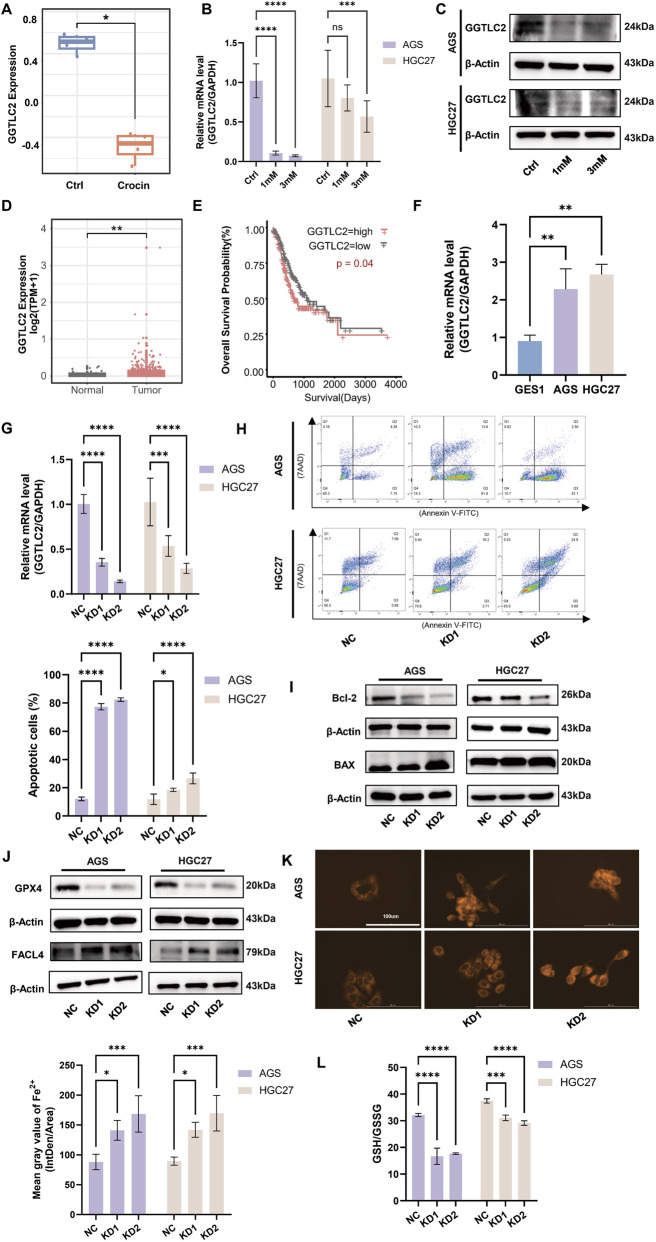
GGTLC2 is highly expressed in GC and is associated with a poor prognosis. **(A)** Gene chip analysis indicates that GGTLC2 expression is downregulated in the crocin treatment group compared to the control group. **(B)** The qPCR results indicate that the expression of GGTLC2 is downregulated following treatment with crocin. **(C)** The WB results indicate that the GGTLC2 level is downregulated following treatment with crocin. **(D)** The analysis of data from the TCGA database indicates that the expression level of GGTLC2 is downregulated in GC tissues. **(E)** Survival analysis shows that high GGTLC2 expression correlates with a poor prognosis. **(F)** qPCR results indicate that GGTLC2 is highly expressed in AGS and HGC27 compared to GES1. **(G)** Knockdown of GGTLC2 was achieved through lentiviral transfection, followed by validation using qPCR. **(H)** Flow cytometry revealed an increase in apoptotic cells following GGTLC2 knockdown. **(I)** WB analysis demonstrated that GGTLC2 knockdown significantly downregulated Bcl-2 expression while concurrently upregulating BAX levels. **(J)** WB analysis demonstrated that GGTLC2 knockdown significantly downregulated GPX4 expression while concurrently upregulating FACL4 levels. **(K)** After knocking down GGTLC2, the expression of Fe2+ is upregulated. **(L)** After knocking down GGTLC2, the expression of GSH/GSSG is reduced. *p < 0.05, **p < 0.01, ***p < 0.001, and ****p < 0.0001. “ns” indicates no significa.

**FIGURE 6:**
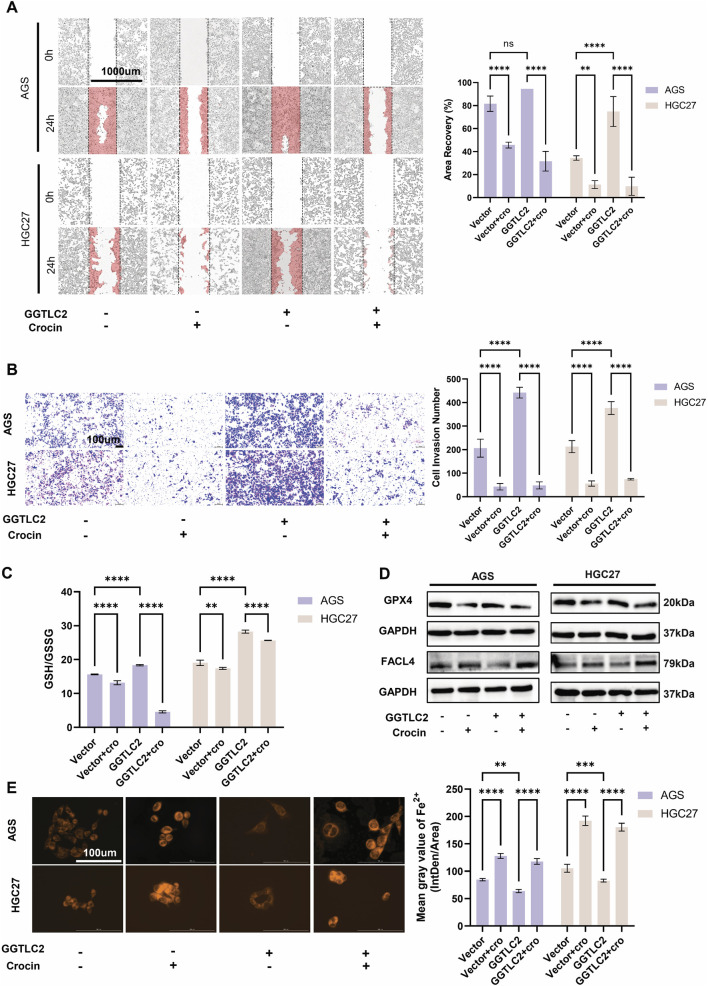
Crocin inhibits the invasion and migration of GC cells by targeting GGTLC2 and promotes ferroptosis. **(A)** Wound healing assays indicate that the OE group’s migration rate increased relative to the Vector group, but both groups decreased after crocin treatment (at IC50 concentration). **(B)** Transwell results indicate that the number of invasive cells in the OE group is significantly increased compared to the Vector group, which is reduced after crocin treatment. **(C)** Compared to the Vector group, the GSH/GSSG ratio in the OE group is significantly elevated; after crocin treatment, the GSH/GSSG ratios in both groups are downregulated. **(D)** WB analysis revealed that the OE group exhibited higher basal GPX4 expression than the Vector group, but crocin treatment downregulated GPX4 in both groups. Conversely, FACL4 expression was lower in the OE group at baseline, while crocin treatment upregulated FACL4 in both groups. **(E)** The OE group exhibits lower levels of Fe2+ compared to the Vector group; however, crocin treatment increased Fe2+ accumulation in both groups. *p < 0.05, **p < 0.01, ***p < 0.001, and ****p < 0.0001. “ns” indicates no significa.

For Figure 3E (GPX4 protein in HGC27 cells) and Figure 4J (FACL4 protein in AGS cells), the β-Actin internal reference blots were mistakenly imported from unrelated experimental groups during figure layout. This error stemmed from an incorrect saving path while capturing numerous WB images for various groups during the experiment. The target protein bands of GPX4 and FACL4 remain authentic, original experimental data from their respective cell groups.

In the wound-healing panels of HGC27 cells (Figure 6A): the 0 h micrograph of the GGTLC2^−^CROCIN^-^ group was accidently replaced with an image captured from the second parallel replicate of the same group; the 24 h micrograph of the GGTLC2^+^CROCIN^−^ group was incorrectly selected from another duplicate well of the same experimental group. This mistake occurred during centralized classification and screening of numerous replicate microscopic images for figure composition.

We have prepared revised versions of Figure 3E, Figure 4J and the affected wound-healing panels (Figure 6A), in which all incorrect images have been replaced with the matched original raw images from the corresponding sample batches or designated experimental wells. Crucially, all migration rate calculations and subsequent statistical analyses throughout these experiments were performed using the original, valid raw data of the corresponding experimental groups. Therefore, the above-mentioned graphical selection mistakes do not alter any quantitative results, statistical significances or core conclusions of the published study. The corrected Figures 3,4,6 appear below.

The original article has been updated.

